# Social media’s traps affecting mental health among young adults

**DOI:** 10.1192/j.eurpsy.2023.1893

**Published:** 2023-07-19

**Authors:** A. H. I. Abu Shehab, L. Luca, S. Trifu, I. Udriştoiu, D. C. Voinescu, A. Ciubară

**Affiliations:** 1 Psychiatry, “Elisabeta Doamna” psychiatry hospital, Galati; 2 Psychology, University of Medicine and Pharmacy “Grigore T. Popa”, Iasi; 3 Psychology, Carol Davila University of Medicine and Pharmacy, Bucharest; 4 Psychiatry, University of Medicine and Pharmacy of Craiova, Craiova; 5 Rheumatology; 6 Psychiatry, „Dunărea de Jos” University of medicine, Galati, Romania

## Abstract

**Introduction:**

Due to technological advancements, a growing number of tasks can now be accomplished with the help of a screen. However, there may be repercussions to leading a lifestyle dominated by digital screens. Our study determines an association between problematic social media use and an increase in the incidence of negative mental health outcomes.

**Objectives:**

This paper is a presentation of the mental health issue of young adults related to the use of social media.

**Methods:**

Analysis of user behavior.

**Results:**

The frequency of using these networks, the length of time spent in front of the screens, the way these platforms influence the daily activities of the users, the emotional states felt by them and the possible connection between the use of the Internet and face-to-face communication remain real challenges for mental health specialists.

**Conclusions:**

Preventing the occurrence of mental health disorders among young adults in the context of the development of technology remains a topical issue.

**Disclosure of Interest:**

None Declared

